# Intracellular amyloid hypothesis for ultra‐early phase pathology of Alzheimer's disease

**DOI:** 10.1111/neup.12738

**Published:** 2021-04-20

**Authors:** Hitoshi Okazawa

**Affiliations:** ^1^ Department of Neuropathology, Center for Brain Integration Research Tokyo Medical and Dental University Tokyo Japan

**Keywords:** expansion of neurodegeneration, high mobility group box 1 (HMGB1), intracellular amyloid hypothesis, transcriptional repression‐induced atypical cell death (TRIAD), Yes‐associated protein (YAP)

## Abstract

Using a new marker of necrosis, pSer46‐MARCKS, which was identified by comprehensive phosphoproteome analysis as a phosphoprotein changed before appearance of extracellular amyloid aggregation, we discovered that neuronal necrosis occurs much earlier in Alzheimer's disease pathology than previously expected. The necrosis is induced by intracellular amyloid accumulation that deprives a critical effector molecule, Yes‐associated protein (YAP), in the Hippo signaling pathway that is essential for cell survival, similarly to TRIAD necrosis observed in transcriptional repression and in other neurodegenerative diseases such as Huntington's disease. The initial TRIAD necrosis due to the intracellular amyloid releases HMGB1 into extracellular space and induces cluster of secondary necrosis around the primary necrotic neurons. Finally, the cluster grows into extracellular amyloid plaque. Inhibition of HMGB1 by anti‐HMGB1 antibody prevents expansion of neurodegeneration. Administration even after onset significantly ameliorates the cognitive decline of Alzheimer's disease model mice. Our results present a new theory of Alzheimer's disease pathology, which can be referred to as the “intracellular amyloid hypothesis".

## INTRODUCTION

The amyloid hypothesis, which was proposed by Professors Hardy and Allsop^1^ nearly 30 years ago, has been critical in the research on the pathogenesis of Alzheimer's disease (AD) and in developing appropriate therapeutics. The amyloid hypothesis proposes that the amyloid beta peptide (Aβ) aggregation (senile plaque) in the “extracellular space” of the human brain is at the top of pathological cascade and that removal of the “extracellular Aβ” should ameliorate and/or reverse the AD pathology as a whole and improve the symptoms of AD patients.

Unfortunately, various anti‐Aβ antibodies with different interaction properties for monomers, oligomers, and polymers of Aβ developed by big pharmaceutical companies have not succeeded in improving the symptoms of AD patients, even though, judging from PET studies, most of such anti‐Aβ antibodies have successfully reduced the Aβ burden in patients’ brains.^2^, ^3^, ^4^, ^5^ In addition, pathological studies of patients receiving active immunization with Aβ peptide who did not reveal side effects, such as severe encephalitis, revealed clearance of Aβ in the brain, while the clinical records of such patients did not support improvement of the symptoms.^6^

The results of three clinical Phase III studies on aducanumab, the last hope of anti‐Aβ antibodies, are now under review by the Food and Drug Administration (FDA), as of December 2020, while on November 6, 2020, the FDA Peripheral and Central Nervous System Drugs Advisory Committee announced that they did not support evidence of the effectiveness of aducanumab for the treatment of Alzheimer's disease in three clinical studies: Study 302 (EMERGE), Study 301 (ENGAGE), and Study 103 (PRIME) (Yes: 0, No: 10, Uncertain: 1).^7^

One explanation for the ineffectiveness of anti‐Aβ antibodies was that the intervention for patients with mild cognitive impairment (MCI) or early‐stage AD in clinical studies was too late. Based on this view, intervention for pre‐onset people who will have AD later should be successful. However, early in 2020, a DIAN study on solanetumab for familial AD patients before the onset of symptoms did not reveal positive results.^8^ However, all the previous results from human studies urged pharmaceutical companies and leading researchers to shift their target from Aβ aggregates to other substances. One such non‐Aβ‐aggregate target is Tau. However, the Tau antibody has also been unsuccessful in human clinical studies of AD and progressive supra‐nuclear palsy (PSP) patients.^9^, ^10^, ^11^

## FROM COMPREHENSIVE ANALYSES TO A NEW “INTRACELLULAR AΒ HYPOTHESIS”

We have not been interested in whether or not the amyloid hypothesis is true but simply in pursuing an understanding of AD pathogenesis and development of AD therapeutics based on comprehensive analyses that could exclude bias of prejudices. From 2010, we started comprehensive phosphoproteome analyses of brain tissues from model mice and human patients with polyglutamine diseases (Huntington's disease and cerebellar ataxia)^12^ and then applied the method to AD to find several proteins whose phosphorylation changed before extracellular Aβ aggregation such as MARCKS (Myristlated Alanine‐Rich C‐kinase substrate).^13^ Our results clearly indicated that the “ultra‐early‐stage pathology of AD” is initiated before the appearance of extracellular Aβ aggregation when nothing should occur according to the amyloid hypothesis.

Furthermore, we discovered that HMGB1‐TLR4 signal triggers MARCKS phosphorylation at Ser46.^14^ HMGB1 is known to be released from cells under necrosis. When HMGB1 concentration was examined, it was higher in patients at the stage of mild cognitive impairment (MCI) than in patients at full AD stage.^15^ Therefore, we used pSer46‐MARCKS as a new marker for “ultra‐early‐stage pathology of AD” and found that ultra‐early‐stage necrosis, which was not detected by other previous methods, occurs actively in multiple types of AD mouse models, such as 5xFAD and amyloid precursor protein‐knock in (APP‐KI) mice before the appearance of extracellular Aβ aggregation.^15^

Moreover, we analyzed in detail the relationship between the ultra‐early‐stage necrosis and intracellular Aβ accumulation by using time‐lapse imaging of iPSC‐derived neurons and by time‐lapse imaging of living cortical neurons in mouse brains with two‐photon microscopy.^15^ The analyses revealed that intracellular Aβ interacts with Yes‐associated protein (YAP), impairs the YAP function to promote cell viability, and, finally, induces necrosis.^15^ Residual intracellular Aβ remains in the extracellular space and becomes the seed for growth to Aβ plaques.^15^

Since our group first reported the existence of intracellularly accumulated Aβ in cortical neurons of human familial AD patients with a presenilin 1 (PS1) gene mutation and in AD model mice,^16^ an increasing number of published studies have confirmed the intracellular accumulation of Aβ,^17^, ^18^ but its function has remained unclear. Our study finally elucidated the pathological function of intracellular Aβ, revealed the relevance to new pathogenic molecules such as HMGB1, MARCKS, and YAP, and established the “intracellular Aβ hypothesis.”^15^

## PHOSPHOPROTEINS CHARACTERIZING THE ULTRA‐EARLY‐STAGE PATHOLOGY OF AD

Our group performed comprehensive phosphoproteome analyses with four types of AD mouse models (mutant PS1‐Tg, mutant PS2‐Tg, mutant APP‐Tg, and 5xFAD) and with postmortem human AD brains. Cerebral cortex tissues were sampled at one, three, and six months of age, and we confirmed with multiple types of anti‐Aβ antibodies that amyloid plaques were not present at one month of age and appeared from three months of age.^13^ Using six types of behavioral tests, including the light–dark box test, the rotarod test, the open field test, the Morris water maze, the elevated plus maze, and the fear conditioning test, no mouse model showed abnormalities before six months of age.^13^ Based on our standard that 60 000 to 100 000 peptides and 700 to 1500 proteins were identified at more than 95% reliability, we identified that the phosphorylation of three proteins (MARCKS, MarcksL1, and SRRM2) was commonly changed across AD mouse models.^13^ This indicates that although the results were obtained from AD mouse models, certain molecular changes occur before extracellular Aβ aggregates are detected by immunohistochemistry (at the level more sensitive than amyloid PET for human patients), which is obviously contradictory to the amyloid hypothesis.

## PSER46‐MARCKS, AN ULTRA‐EARLY PHASE PATHOLOGY MARKER, RECOGNIZES DEGENERATIVE NEURITES

**Fig. 1:**
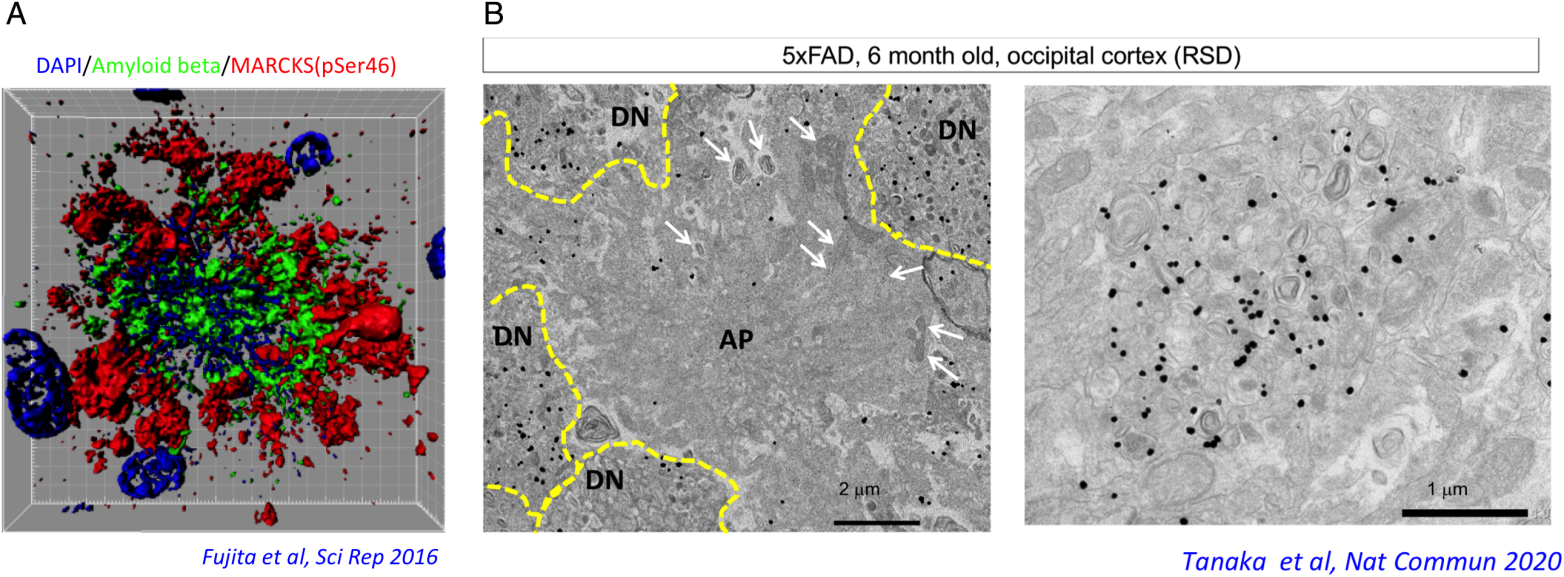
Necrosis due to intracellular accumulation surrounded by pSer46‐MARCKS‐positive degenerative neurites.

We next selected four phosphorylation sites of MARCKS from a number of phosphorylation sites under the conditions that they were changed at one month of age and that they were also changed in postmortem human AD brains.^14^ We also generated antibodies against four phosphorylation sites and performed immunohistochemistry. Surprisingly, the antibody against pSer46‐MARCKS revealed stains whose pattern was similar to extracellular amyloid plaques or senile plaques at low magnification. High magnification revealed dot‐like or patch‐like stains of pSer46‐MARCKS, which are distinct from but surrounding extracellular amyloid plaques or senile plaques.^14^ Immunoelectron microscopic analysis confirmed that pSer46‐MARCKS is localized to degenerative neurites surrounding extracellular amyloid plaques.^14^ Intriguingly, a destroyed nucleus or nuclei were frequently stained at the center of the extracellular amyloid plaque^14^ (Fig. 1).

## HMGB1 RELEASED FROM NEURONAL NECROSIS INDUCES NEURITE DEGENERATION

At this point, we came to the hypothesis that neuronal necrosis occurs at the site where extracellular amyloid plaque is formed later and that some damage associated molecular patterns (DAMPs) molecules influence neurites connecting or neighboring the necrotic neuron and there induce cell signaling, leading to pSer46‐MARCKS that degenerate neurite and dendritic spines.^14^ MARCKS functions as a connecter linking the intracellular actin network to the cell membrane, which is essential for maintaining protrusion or indentation of the cell membrane. It has also been reported that the postsynaptic membrane protrusion, the dendritic spine, becomes unstable when MARCKS protein is phosphorylated.^19^

We examined the hypothesis by adding DAMPs molecules such as Aβ, tau, and HMGB1 and found that HMGB1 induces pSer46‐MARCKS most efficiently. Aggregated Aβ has no effect and Aβ oligomer has a weaker effect than HMGB1 on MARCKS phosphorylation at Ser46.^14^ Moreover, various experiments revealed that Toll‐like receptor 4 (TLR4) functions as a receptor for HMGB1 and induces MARCKS phosphorylation at Ser46 via protein kinase C activation.^14^

## ANTI‐HUMAN HMGB1 HUMAN MONOCLONAL ANTIBODY HAS A THERAPEUTIC EFFECT ON AD MODEL MICE

Moreover, we generated a new mouse monoclonal antibody against HMGB1 and revealed that administration of the antibody before the onset ameliorated cognitive impairment at the time point from onset and one month later.^14^ Therefore, we again generated anti‐human HMGB1 human monoclonal antibody, which has a high affinity to human HMGB1, cross‐reacts with mouse HMGB1, suppresses the interaction between mouse/human HMGB1 and TLR4, and revealed that, even after the onset, administration of the anti‐human HMGB1 human monoclonal antibody rescued the cognitive impairment of the AD model mice (Okazawa, Patent Application: 20190211092 16/324192).

## NEURONAL NECROSIS OCCURS AT ULTRA‐EARLY PHASE BEFORE EXTRACELLULAR AMYLOID AGGREGATION

**Fig. 2:**
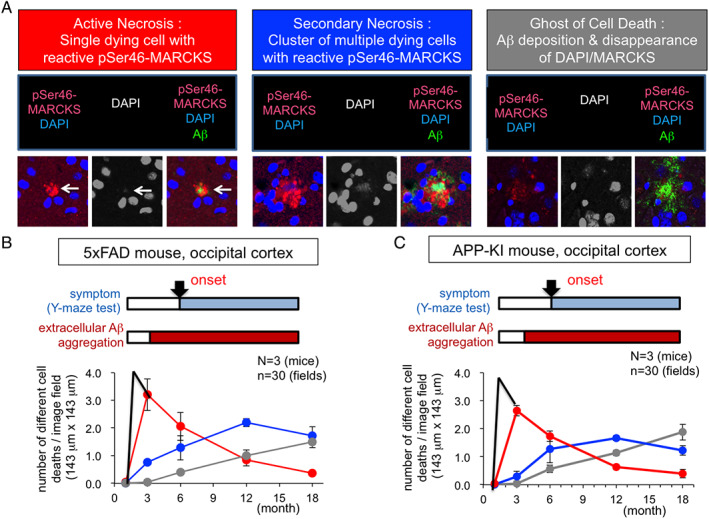
Neuronal necrosis at ultra‐early stage.

We next utilized pSer46‐MARCKS as a marker for necrosis. Neurons under ongoing necrosis accompany reactive degeneration of surrounding neurites, which are stained by pSer46‐MARCKS, and such neurons possess nuclei with weak DAPI signals. Using the criteria, we quantified the frequency of active necrosis in the cerebral cortex tissues of 5xFAD and mutant APP‐KI mice every month from birth and revealed that active necrosis reached peak frequency at two months of age before the appearance of extracellular Aβ aggregates but continued to 18 months of age.^15^ Thereafter, secondary necrosis, a cluster of necrosis of multiple neurons around a neuron that had undergone the initial necrosis, increased in frequency. Finally, the temporal transition of three pathological changes was observed; stains of DAPI and pSer46‐MARCKS weakened, and the image in which only extracellular amyloid remained was increased from the very end^15^ (Fig. 2). The existence of neuronal necrosis before the appearance of extracellular amyloid aggregates has not been expected previously; thus, the scheme presented from our studies might be able to explain why human clinical studies targeting extracellular amyloid aggregates have failed up to now.

## EXTRACELLULAR AMYLOID AGGREGATION IS THE RESULT OF NEURONAL NECROSIS INDUCED BY INTRACELLULAR AMYLOID ACCUMULATION

Collectively, our analyses of active necrosis in two types of AD mouse models with a new marker pSe46‐MARCKS indicated that accumulation of intracellular amyloid causes neuronal necrosis, leading to extracellular amyloid aggregation. In other words, that extracellular amyloid aggregation is the result of neuronal necrosis caused by intracellular amyloid.^15^

To examine this new concept, we differentiated neurons from iPS cells carrying APP mutation generated by genome editing and investigated necrosis of the iPSC‐derived neurons by time‐lapse imaging. Consequently, we found that accumulation of intracellular amyloid is followed by abnormal expansion of ER, causing necrotic rupture of cells.^15^ In parallel, we observed living cortical neurons *in vivo* by two‐photon microscopy. Intracellular amyloid accumulation expands ER to induce necrosis and intracellular amyloid remains in the extracellular space after cell death.^15^

## DEVELOPMENT OF THERAPEUTICS BASED ON MOLECULAR MECHANISM OF NECROSIS BY INTRACELLULAR AMYLOID

Finally, we investigated the molecular mechanism of active necrosis. Active necrosis is morphologically characterized by extreme expansion of ER, and it is identical to the feature of transcriptional repression‐induced atypical cell death (TRIAD) related to YAP that we reported in transcriptional repression^20^ and in Huntington's disease pathology.^21^, ^22^, ^23^ Therefore, we analyzed the relationship between YAP dynamics and cell death using time‐lapse imaging of iPSC‐derived neurons carrying APP mutation, and found that intracellular amyloid traps YAP in ER and the resultant decrease of YAP in the nucleus induces necrosis, similarly to TRIAD.^15^

Therefore, we examined the therapeutic effect of adeno‐associated virus (AAV)–YAP rescuing the impairment of YAP function and revealed that AAV–YAP not only rescued cognitive decline significantly but also decreased extracellular amyloid aggregation of AD model mice remarkably.^15^ Consistently, we found that siRNA‐mediated knockdown of YAP induces TRIAD.^15^ All the results collectively indicated that necrosis at the ultra‐early phase of AD pathology is exactly TRIAD.^15^

**Fig. 3:**
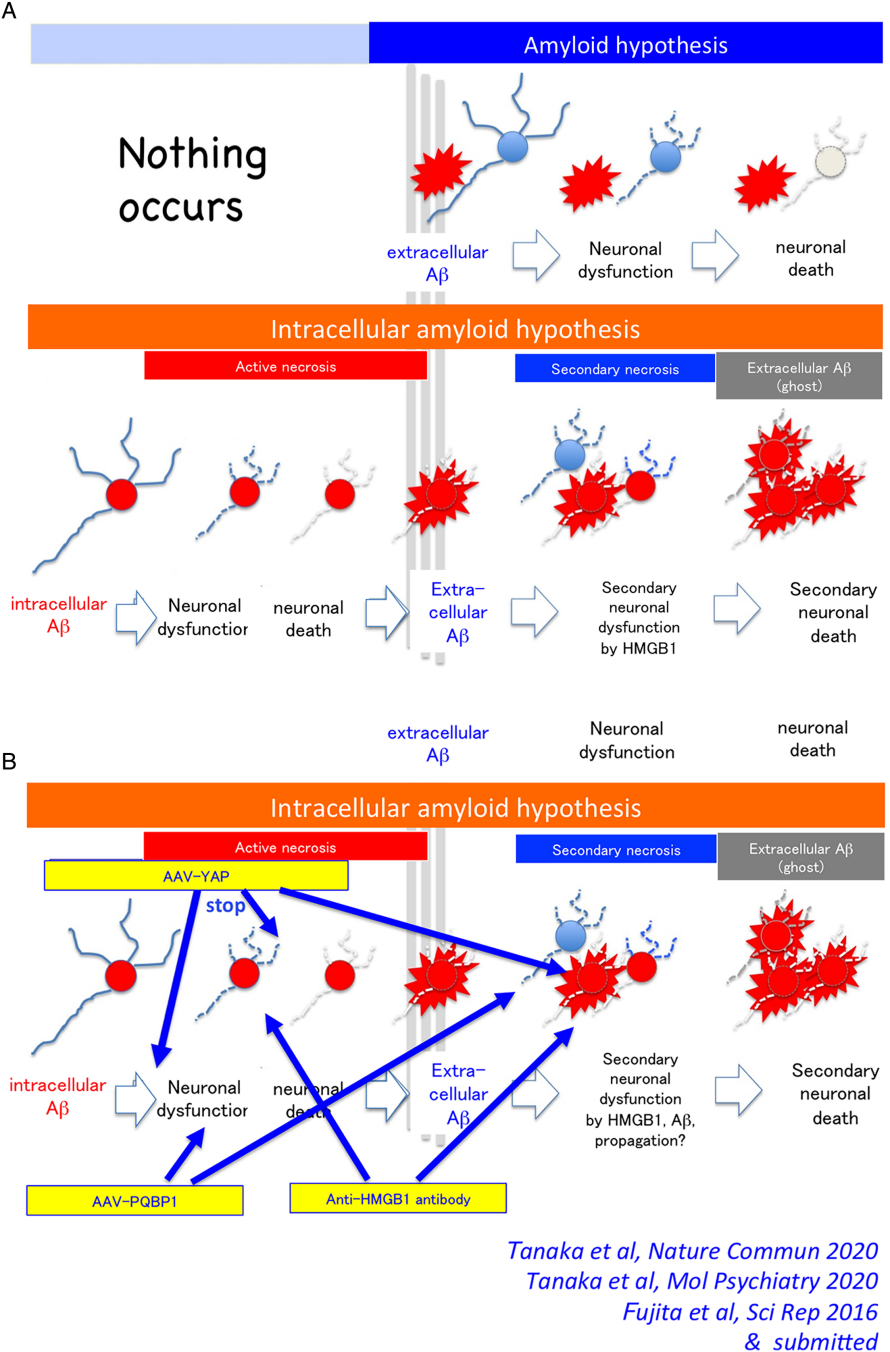
Intracellular amyloid hypothesis and therapeutic approaches targeting the ultra‐early stage pathology before extracellular amyloid aggregation.

Our data‐driven research, which is independent of the existing AD hypothesis, presented an obviously distinct view for AD pathology. Extracellular amyloid aggregation is the result of TRIAD necrosis caused by intracellular amyloid accumulation. Neuronal degeneration spreads to surrounding neurons due to DAMPs such as HMGB1 being released from original necrosis (there remains a possibility that amyloid oligomers also play such a function), which accelerates the AD pathology. The phenomenon at a glance seems homologous to prionoid transmission of degenerative proteins, while it is clearly distinguished because DAMPs mediate the expansion of degeneration. Inhibition of the degeneration expansion by anti‐HMGB1 antibody and suppression of necrosis by AAV‐YAP both have therapeutic effects, and they are complementary and synergic as therapeutics targeting sequential phases in the ultra‐early phase pathology of AD (Fig. 3).
